# Oocyte diameter predicts the maturation rate of human immature oocytes collected ex vivo

**DOI:** 10.1007/s10815-022-02602-0

**Published:** 2022-09-10

**Authors:** S. E. Pors, D. Nikiforov, J. Cadenas, Z. Ghezelayagh, Y. Wakimoto, L. A. Z. Jara, J. Cheng, M. Dueholm, K. T. Macklon, E. M. Flachs, L. S. Mamsen, S. G. Kristensen, C. Yding Andersen

**Affiliations:** 1 Laboratory of Reproductive Biology, The Juliane Marie Centre for Women, Children and Reproduction, Copenhagen University Hospital and Faculty of Health and Medical Sciences, University of Copenhagen, 2100 Copenhagen, Denmark; 2 Department of Obstetrics and Gynecology, Hyogo College of Medicine, Nishinomiya, Hyogo, 663-8501 Japan; 3 People’s Hospital of Guangxi Autonomous Region, Nanning, 530000 China; 4 The Fertility Clinic, Aarhus University Hospital, Skejby, 8200 Aarhus, Denmark; 5 Juliane Marie Centre for Women, Children and Reproduction, The Fertility Clinic, Copenhagen University Hospital and Faculty of Health and Medical Sciences, University of Copenhagen, 2100 Copenhagen, Denmark; 6 Department of Occupational and Environmental Medicine, Bispebjerg University Hospital, 2200 Copenhagen, Denmark

**Keywords:** Oocyte maturation, Oocyte diameter, Fertility preservation, Ovarian tissue cryopreservation, In vitro maturation

## Abstract

**Purpose:**

To study the impact of oocyte diameter and cumulus cell mass on the potential for final maturation of immature human oocytes in vitro.

**Methods:**

Immature oocytes (*n* = 1563) from 75 women undergoing fertility preservation by ovarian tissue cryopreservation (14–41 years) were collected. After preparation of the ovarian cortex for freezing, immature oocytes were collected from the surplus medulla. After collection, IVM was performed according to standard published methods. The mass of cumulus cell surrounding the immature oocyte was grouped according to size. After IVM, each oocyte was photographed, measured, and the diameter was calculated as a mean of two perpendicular measurements.

**Results:**

The diameter of the oocytes ranged from 60 to 171 µm with a mean of 115 µm (SD:12.1) and an interquartile range from 107 to 124 µm. The oocyte diameter was positively associated with a higher incidence of MII (*p* < 0.001). MII oocytes had a significantly larger mean diameter than MI, GV, and degenerated oocytes. The size of the cumulus cell mass was significantly associated with the MII stage (*p* < 0.001) and larger oocyte diameter (*p* < 0.001). The results further confirm that the diameter of the fully grown oocyte is reached relatively early in human follicular development and that the factors governing oocyte maturation in vitro are connected to the surrounding cell mass and the oocyte.

**Conclusion:**

The diameter of the oocyte is a highly determining factor in the nuclear maturation of the human oocyte during in vitro maturation, and the size of the cumulus cell mass is closely positively associated with a larger diameter.

## Introduction

In women, follicle growth and maturation from the primordial stage to the fully developed preovulatory follicle last up to half a year [1–3]. During this process, the human oocyte grows from a diameter of 35 µm in the primordial follicle [1] to a diameter usually exceeding 100–110 µm when an antrum starts to form at a follicular diameter of around 250 µm [2]. At ovulation, the oocyte has reached its final diameter of around 110–120 µm [3]. However, the ability to resume meiosis is only fully developed at the time of ovulation, where the oocyte responds to the midcycle surge of gonadotropins by advancing from the germinal vesicle stage (GV) to the metaphase II stage (MII) of meiosis (i.e., nuclear maturation) and undergoes final cytoplasmatic maturation including redistribution of organelles in order to support further development [4].

Assisted reproduction is performed in most mammalian species, except humans, by aspirating immature oocytes from ovaries ex vivo, maturing them to the MII stage (in vitro maturation, IVM), and subsequently performing standard in vitro fertilization (IVF). This is an accepted method by which a massive number of healthy offspring have been produced. Interestingly, a number of studies of oocytes from mammalian species, including dogs, rats, rhesus monkeys, goats, and mice, have identified a minimal oocyte diameter, specific for each species, which is required for the oocyte to sustain nuclear maturation following IVM [5–10]. In humans, only a few studies have investigated whether a similar lower threshold of oocyte diameter is needed to sustain nuclear maturation and whether an increased diameter is associated with a higher frequency of MII transition. Durinzi and coworkers [11] were the first to report that meiotic resumption of human oocytes, collected from unstimulated ovaries, was influenced by oocyte diameter. This has subsequently been confirmed using oocytes obtained from whole ovaries of women receiving no ovarian stimulation [12], oocytes collected from women suffering from PCOS with or without preceding stimulation [13, 14], and oocytes collected from patients undergoing ovarian stimulation and intracytoplasmic sperm injection (ICSI) [14]. Studies have also suggested that an increased diameter of human oocytes at the GV stage, collected after ovarian stimulation is linked to reduced transcriptional activity and increased competence for maturation [15–17]. Although the number of oocytes included in these studies is limited, the data suggest that oocyte diameter reflects the capacity for human oocytes to sustain nuclear maturation. In the current study, a large number of oocytes were collected ex vivo from whole ovaries in connection with ovarian tissue cryopreservation (OTC) for fertility preservation and subsequently subjected to IVM. The present study aimed to explore whether the oocyte diameter and size of the cumulus-oocyte complex (COC) were associated with the MII rate of oocytes undergoing IVM obtained from unstimulated women.

## Materials and methods

### Patients and collection of oocytes

From 75 patients, whole ovaries were obtained for fertility preservation at two different clinics. The ovarian cortex was cryopreserved at the Laboratory of Reproductive Biology, University Hospital of Copenhagen, Denmark. None of the patients had received any type of ovarian stimulation with exogenous hormones prior to the procedure. The ethical committee of the municipalities of the Capital Region of Denmark approved the project (H-2–2011-044), and all the patients signed an informed consent form prior to the surgical procedure, in which they consented to donate their surplus ovarian tissue including immature oocytes for research. At arrival, the weight of the trimmed ovary equal to the ovarian volume was monitored [18, 19]. After procurement of the cortex, the surplus medulla tissue was examined for the presence of immature COC released from small antral follicles with diameters less than 3 mm. Oocyte collection and IVM have been described [20, 21] with all culture media supplemented with the growth factor midkine.

### Classification and measurement of cumulus-oocyte complexes

The sizes of the cumulus cell mass surrounding the immature oocyte were grouped as naked oocytes with no cumulus cells surrounding the oocytes, small cumulus-oocyte complexes (S-COCs) with 3–10 layers of cumulus cells, and large cumulus-oocyte complexes (L-COCs) with more than 10 layers of cells. The diameter of the cumulus cells was photographed and measured before IVM using the AxioVision software (SE64 Rel.4.9.1).

### Measurement of oocyte diameter

After IVM, oocytes were denuded, and photos of all oocytes were taken using a Carl Zeiss microscope and the AxioVision software (SE64 Rel.4.9.1). The diameter of the oocytes was obtained by calculating the average of two measurements perpendicular to one another assessed from the inner side of the zona pellucida. Thus, the zona pellucida was not included.

### Statistical analysis

The frequency of maturation for immature oocytes to MII was modeled as a mixed logistic regression with maturation to MII (yes/no) as an outcome and transport of ovary (yes/no), COC size (naked, small, and large), diagnosis (breast cancer/any other condition), age of the patient, the weight of the ovary, and oocyte diameter (linear effect) as covariates. The random effect of the patient was included. Oocyte diameter as an outcome was modeled using COC diameter, diagnosis, maturation stage of the oocytes (degenerated, GV, MI, and MII), age of the patient, the weight of the ovary, and the random effect of the patient. All analysis was done in R (version 3.4.3).

## Results

### Patient characteristics

The diagnosis of patients were breast cancer (*n* = 33), hematological cancer (*n* = 11), gastrointestinal cancer (*n* = 7), central nervous system cancer (*n* = 5), benign disease (*n* = 6), other (*n* = 6), osteosarcoma and sarcoma (*n* = 5), and gynecological cancer (*n* = 2). The mean age was 26.7 years (range: 14–40.9 years). The mean weight of the ovaries was 8.2 g (SD: 0.09, range: 2.4–19.8 g), corresponding to a volume of 8.2 ml [18].

### Collected oocytes and COCs

A total of 1563 oocytes with a recorded diameter were included. After IVM, 489 (31%) of the oocytes were MII, 307 (20%) were MI, 469 (30%) were GV, and 298 (19%) were DEG. Of the 1563 oocytes, 618 (40%) were found in L-COCs, 597 (38%) in S-COCs, and 348 (22%) oocytes were naked. A total of 310 COCs was measured; of these, 149 were L-COCs with a mean diameter of 493 µm (range 223.6–834.4 µm), and 161 were S-COC with a mean diameter of 249 µm (range 161–536 µm). The diameter of L-COCs was significantly larger compared to the diameter of S-COCs (*p* < 0.002).

### Oocyte diameter and stage of maturation

The oocyte diameter ranged from 60 to 171 µm with a mean of 115 µm (SD:12.1). The interquartile range was from 107 to 124 µm revealing that 75% of the oocytes had a diameter larger than 107 µm. The oocyte diameter was positively associated with a higher incidence of MII development (*p* < 0.001). Oocytes with different stages of maturation (GV, MI, or MII) after IVM had significantly different diameters (*p* < 0.0001) with oocytes reaching the MII stage having a significantly increased diameter as compared to the MI and GV stages (Table 1).

**Table 1:** Oocyte diameter and maturation in relation to COC size for 1563 oocytes collected ex vivo in connection with ovarian cryopreservation for fertility preservation

	Total		Large COC		Small COC		Naked oocytes	
	Oocytes (*n*)	Oocyte diameter µm (mean ± SD)	Oocytes (*n*)	Oocyte diameter µm (mean ± SD)	Oocytes (*n*)	Oocyte diameter µm (mean ± SD)	Oocytes (*n*)	Oocyte diameter µm (mean ± SD)
Total	1563	115 ± 12.1	618	118 ± 10.5^**^	597	113 ± 11.4^*^	348	112 ± 14.8^*^
MII	489	123 ± 9.7^a^	267	123 ± 8.8	157	122 ± 9.9	65	127 ± 11.6
MI	307	115 ± 8.8^b^	129	116 ± 8	133	115 ± 8.4	45	113 ± 11.5
GV	469	108 ± 9.7^c^	178	111 ± 8.4	207	107 ± 9.2	84	105 ± 11.7
Degenerated	298	111 ± 13.7^d^	44	117 ± 14.2	100	111 ± 11.8	154	110 ± 14.4
Maturation rate (%)	31		43		26		19	

The cumulus cell mass surrounding the immature oocytes was grouped as naked oocytes with no cumulus cells surrounding the oocytes, small COCs with 3–10 layers of cumulus cells, and large COCs with more than 10 layers of cells

Statistically different numbers are marked with different letters or numbers of asterisks (^*^)

### Oocyte diameter and COC size

**Fig. 1:**
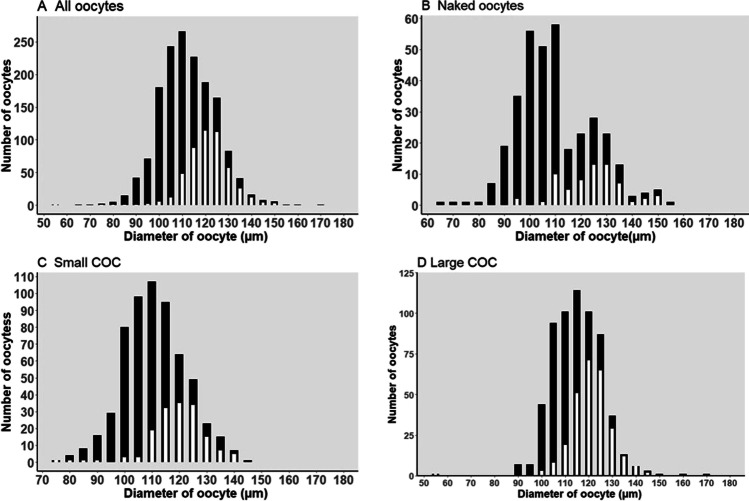
Number of oocytes collected in connection to ovarian cryopreservation according to diameter measured after IVM. Black bars: total number of oocytes at a given diameter. White bars: number of oocytes matured to MII at a given diameter. The diameter of the oocytes ranged from 59.9 to 161.3 µm with a mean of 114.9 µm (SD:12.1) with an interquartile range from 107 to 124 µm. The cumulus cell mass surrounding the immature oocytes was grouped as naked oocytes with no cumulus cells surrounding the oocytes, small COCs (Fig. 1C) with 3–10 layers of cumulus cells, and large COCs (Fig. 1D) with more than 10 layers of cells

**Fig. 2:**
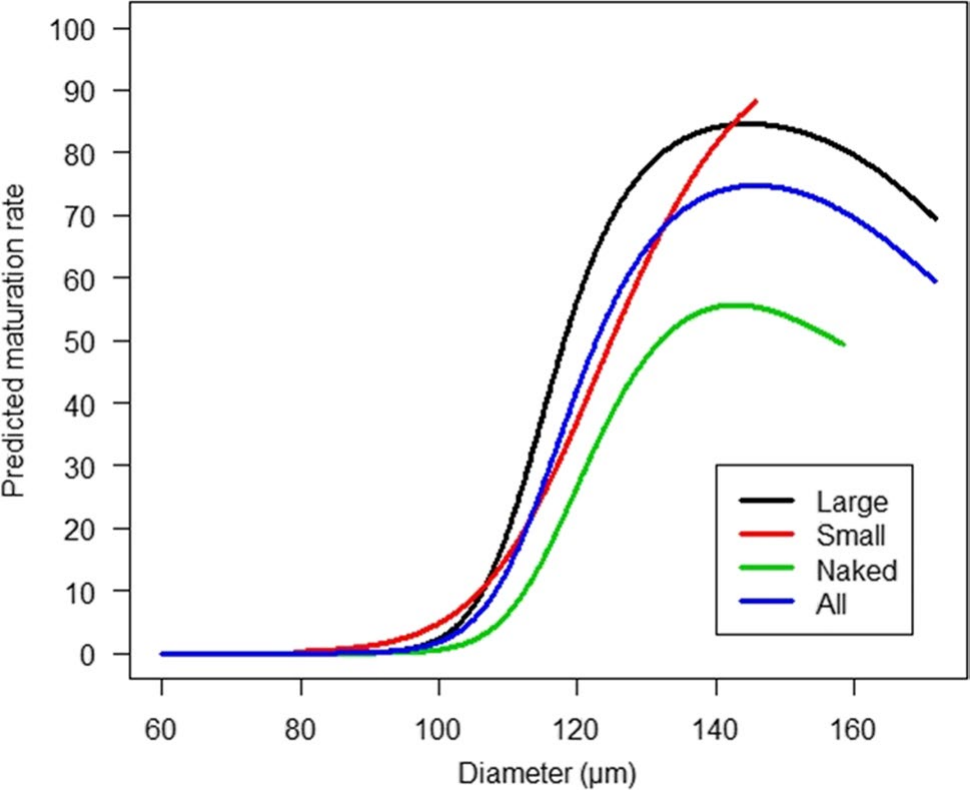
Predicted maturation rate depending on oocyte diameter and size of COC. The prediction was done in a linear mixed model with the patient as a random effect. The oocytes were grouped as naked oocytes with no cumulus cells attached, small COCs with 3–10 layers of cumulus cells, and large COCs with more than 10 layers of cells

The COC size was significantly associated with MII stage (*p* < 0.001) and larger oocyte diameter (*p* < 0.001) (Table 1). In Fig. 1, the distribution of oocytes within different COC sizes is shown. Figure 2 shows the expected maturation rate of oocytes predicted by the diameter and the maturation rate depending on the different COC sizes. The model predicts that oocytes with a diameter below 100 µm have a 5% chance of reaching MII (7 MII oocytes out of a total of 138 oocytes with a diameter < 100 µm). Oocytes with a diameter of 145 µm in a L-COC had the highest predicted maturation rate (85%). Oocytes with a diameter of 142 µm in S-COCs showed a maximum predicted maturation rate of 88%. Naked oocytes showed a maximum predicted maturation rate of 56%.

### Oocyte diameter and patient characteristics

No association was found between the diameter of oocytes and the age of the patient (*p* = 0.1) or the size of the ovary (*p* = 0.3). The number of women with a diagnosis of breast cancer was sufficiently large to allow a comparison of oocyte diameter with all other diagnoses. However, no significant differences were found overall nor for oocytes with different sizes of COCs (*p* > 0.10).

## Discussion

This study showed a significant positive correlation between oocyte diameter and ability to sustain nuclear maturation measured as MII transition during a 44-h incubation period based on more than 1500 oocytes from small antral follicles obtained from non-stimulated human ovaries.

Furthermore, the number of cumulus cells surrounding the oocyte (i.e., the size of the COC) was of importance for its ability to undergo MII transition. Thus, this study confirms and extends that both oocyte diameter and cumulus mass are essential components of the ability of human oocytes to sustain IVM. These two parameters appear to affect oocyte maturation independently as oocyte diameter is unrelated to the size of the COC. Thus, the fact that the human oocyte reaches its final diameter in relatively small follicles and that cumulus cells can be stimulated to improve capacity for oocyte maturation in vitro [20, 21] enforces that human IVM has the potential to be developed into a clinically relevant alternative also with oocytes from small antral follicles.

Furthermore, this study clearly showed that there is an asynchrony between follicular diameter and oocyte diameter. Oocyte diameter reaches its final dimension, which on average is 110–120 µm in diameter, in relatively small follicles of around just a few millimeters, and does not appear to increase in diameter (or only marginally) during follicular development from small antral follicles until ovulation [22]. This is surprising since it is commonly agreed that oocytes from small antral follicles possess poor developmental competence in terms of MII transition during IVM [23, 24], but the importance of the cumulus cells, which in vitro may be stimulated to induce an accelerated maturation of oocytes as compared to the normal in vivo process, add to the proposition that an effective IVM protocol for fully grown human oocytes deriving from small antral follicles can be developed when more information regarding the hormonal regulation and somatic cells interactions with oocyte maturation is available. In this regard, the current study suggests that it may also be of interest to focus on the mechanisms that promote oocyte growth potentially via stimulation of the cumulus cells.

The oocytes included in our study originated from small antral follicles in the ovaries of unstimulated women and are all from non-macroscopically visible follicles (less than 3 mm in diameter). Data from the present study now substantially increases previous data in both origin and volume (11,12). We found that the average oocyte diameter is 115 µm, which corresponds quite closely to that previously reported and confirms that the oocytes reach their final diameter already at the small antral stage [11, 12, 24]. In our study, we found oocytes undergoing MII transition on average had a diameter of 123 µm (Table 1), and the frequency of MII transition of oocytes below 100 µm was as modest as 5% [7/138].

Oocytes from cattle are often used as a model for the regulation of human oocyte maturation. However, in cattle oocyte and follicle growth continue in parallel until the follicle reaches a diameter of 3 mm [23], which is in contrast to humans where the final oocyte diameter is reached in smaller follicles [22]. After a diameter of 3 mm follicles in cattle may continue until a diameter of 15–20 mm, during which the oocyte remains at a diameter similar to that of a 3 mm follicle (i.e., 120–130 µm) [23]. Bovine oocytes aspirated from slaughterhouse ovaries divided into the following size categories (< 100 µm, 100 to < 110 µm, 110 to < 120 µm, and > 120 µm) showed MII transition with frequencies of 21%, 42%, 76%, and 81%), respectively, for the four groups. It is accepted that as follicle diameter in cattle increases the rate of MII oocytes also goes up until a diameter of 3 mm. Thus, the oocyte developmental competence of bovine oocytes is also very dependent on the diameter, which in this species to some extent reflects the diameter of the follicle from which it derived. The bovine model does therefore not reflect the situation in humans where the final oocyte is reached considerably earlier in follicular development than in cattle. Collectively, the current data mainly including oocytes from follicles with a diameter of less than 3 mm adds new information to the regulation of oocyte maturation in human oocytes.

Human small antral follicles with a diameter of 0.4 to 2.0 mm have been reported to have an atresia rate of 15 to 24% [3]. This rate is considerably lower than in follicles of larger diameters but does suggest that a substantial number of oocytes does not have the capacity to sustain further development. On the other hand, the smaller the follicles from which fully grown oocytes can be isolated, the higher the number of oocytes will be available in the non-stimulated ovaries and can potentially be isolated. In a previous study, we isolated an average of 36 immature oocytes per ovary from 25 consecutive enrolled patients, which, however, included two PCOS patients with a very high number of oocytes, but an average of around 25 oocytes in non-PCOS patients is not unlikely [20, 21]. Such numbers may facilitate a more widespread clinical use in connection with fertility preservation of ovarian cortical tissue, especially if a good reproductive outcome can be demonstrated.

The diameter of the oocytes has been associated with other parameters than maturation. A study by Lazzaroni-Tealdi and co-workers (2015) included diameter as part of an oocyte quality assessment index used to choose the best oocytes in connection with IVF treatment. Here oocyte diameter was the single parameter clearly associated with improved day-3 embryo cell numbers, thus extending the impact of oocyte morphology to also impact embryo formation [25]. However, a study by Romão and coworkers [26] found no significant association between diameter in oocytes collected from follicles in connection with IVM and fertilization and embryo classification. The diameter of oocytes has also been reported to show a higher variability with increasing age and advanced age leads to oocytes with a reduced diameter [27, 28]. However, in the present study, we were unable to document the effect of age on oocyte diameter. It has also been reported that a high body mass index (BMI) reduces the diameter of the oocytes [28, 29]. Unfortunately, we were unable to obtain BMI from the women donating oocytes in this present study.

It is a limitation of the current study that the matured oocytes were not subjected to fertilization to monitor further development, but the Danish authorities do not recognize human IVM using oocytes from small antral follicles as standard treatment to be used in clinical work. Furthermore, it could potentially also have been of interest to focus on other characteristics like for instance partial expansion of cumulus mass (i.e., outer layers vs inner layers of the cumulus). However, this was not included in the original protocol for this project.

Collectively, the present study confirms the oocyte diameter and the somatic cell compartment as being of utmost importance for the human oocyte to undergo MII transition during human IVM. The results further confirm that the diameter of the fully grown oocyte is reached relatively early on in human follicular development and that the factors governing oocyte maturation in vitro are connected to the surrounding cell mass and the oocyte. Taken together, the results of this study suggest further studies to develop the platform for human IVM, as there are ample possibilities to stimulate the somatic cells and, in this way, augment and improve the MII transition.
